# Fiscal Decentralization, Green Technology Innovation, and Regional Air Pollution in China: An Investigation from the Perspective of Intergovernmental Competition

**DOI:** 10.3390/ijerph19148456

**Published:** 2022-07-11

**Authors:** Di Wang, Zhiyuan Zhang, Ruyi Shi

**Affiliations:** 1 School of Economics and Management, China University of Mining and Technology, Xuzhou 221116, China; zhangzhiyuan@cumt.edu.cn; 2 Think Tank of Carbon Neutral and Energy Strategy, China University of Mining and Technology, Xuzhou 221116, China; 3 School of Public Policy and Management, China University of Mining and Technology, Xuzhou 221116, China; 4 School of Safety Engineering, China University of Mining and Technology, Xuzhou 221116, China

**Keywords:** air pollution, fiscal decentralization, green technology innovation, spatial Durbin model

## Abstract

Fiscal decentralization (FD), as an institutional arrangement for the fiscal division between central and local governments, gives local governments the enthusiasm and autonomy to provide public products and services. With the dominance of environmental governance, how local governments can avoid intergovernmental “race to the bottom” issues through green technology innovation (GTI) is a matter of regional green development and continuous improvement of atmospheric environmental quality. Based on a sample of 30 provinces in China from 2003 to 2018, this paper uses the spatial Durbin model (SDM) to examine the relationship between FD, GTI, and regional air pollution and explores their spatial spillover effect and regional heterogeneity from the perspective of intergovernmental competition. The results indicate that the FD and GTI in various provinces had significant and regionally differentiated inhibitory effects on local air pollution. In Western China, due to the regional competition among local governments in terms of economic development, economic development-oriented fiscal expenditures crowd out environmental governance-oriented fiscal expenditures, which has led to the consequence that FD can intensify local air pollution and has a positive spillover effect, but the demonstration effect of green technological innovation can well moderate the effect of FD on air pollution. FD in the eastern region has played a positive role in promoting regional air quality improvement. However, its green technological innovation has not played a positive role in reducing emissions, and it plays a significant negative regulatory role in the emission reduction effect led by FD. Finally, the article puts forward policy recommendations in terms of a fiscal decentralization system, green technological innovation, and performance evaluation mechanism.

## 1. Introduction

Since the reform and opening up, China’s rapid economic growth has been accompanied by excessive energy consumption and rapid environment deterioration [1,2,3]. Facing increasingly prominent environmental pollution, especially the problem of air pollution related to people’s livelihood, the Chinese government has adopted a series of administrative and economic measures to compensate for the failure of market mechanisms in environmental protection [4]. The government’s environmental protection fiscal expenditures have provided several public services through direct investment in environmental governance projects and subsidies for green innovation [5], which have played an essential role in environmental governance. In the *19th National Congress of the Communist Party of China report*, President Xi Jinping explained the relationship between fiscal and taxation system reforms, green technological innovation, and environmental quality. At the same time, he pointed out that it is necessary to establish a central and local fiscal relationship with clear powers and responsibilities, financial coordination, and regional balance to build a market-oriented green innovation system and to rely on appropriate environmental regulatory tools to promote green development to solve prominent ecological problems [6].

Due to the negative externalities of air pollution and the positive externalities of atmospheric environmental governance, regions with low fiscal revenues often have a “free-rider” mentality, thus weakening the function of environmental management to develop the local economy, leading to the crowding out of environmental governance investment, reduction of green technology R&D expenditure, and relaxation of regional environmental regulations, etc. [7]. The atmospheric environment as an “unowned commons” often becomes the victim of a “race to the bottom” among governments, leading to substantial environmental governance costs. Under the current realistic background of intergovernmental competition, regional FD and technological innovation are closely related to the atmospheric environmental quality [8]. As the central government’s principal agent, local governments shoulder the dual mission of developing the local economy and environmental governance. The fiscal decentralization system gives local governments the enthusiasm and autonomy to provide public products and services according to local conditions. However, the reality of intergovernmental competition and different performance evaluation mechanisms determine the different development orientations and decision-making preferences of local governments. Therefore, “competition to the bottom” or “competition to the top” cannot be generalized. Especially under the concept of green development, GTI has a strong demonstration effect in improving the quality of the regional atmospheric environment. Will this effect prompt local governments to improve the expenditure structure of FD, thereby affecting regional atmospheric environmental quality? Therefore, clarifying the influencing mechanism of FD and GTI on regional air pollution can help to optimize the expenditure structure of the FD of local governments as well as guide the green innovation behaviors of polluting enterprises and, ultimately, promote the coordinated development of regional economy and environment.

Many scholars have discussed the impact of fiscal decentralization and intergovernmental competition on air pollution [9]. However, there are still some shortcomings: Firstly, although there is a large amount of literature focusing on the effects of fiscal decentralization on environmental quality [10], much of the literature ignores the characteristics of transboundary air pollution and fail to discuss the spatial spillover effect of FD on air pollution reduction in near-neighboring areas from the perspective of intergovernmental competition [11]. Secondly, in spite of the substantial literature focusing on the influence mechanism among fiscal decentralization, green technological innovation, and environmental quality, most scholars use the overall technical progress or number of general patents to measure the level of GTI, while such indicators are less related to environmental quality and can hardly reflect the green characteristics of technological innovation [12,13,14]; in addition, the adjustment mechanism of GTI on the emission reduction effect of fiscal decentralization has been ignored [7].

Based on China’s strategic arrangement for continuous improvement of environmental quality, this paper reveals the mechanisms of FD and GTI on regional air pollution from the perspective of intergovernmental competition and discusses the effects of FD and GTI on air pollution in terms of the spatial spillover and regional heterogeneity and focuses on the moderating effect of GTI on the pollution emission reduction impact of fiscal decentralization. Different from the existing research, the marginal contributions of this article are as follows: Firstly, this paper uses the number of green technology patents to indicate the level of green technology innovation, which improves the construct validity of the GTI index and objectively reflects the impact of green technology innovation on regional air pollution. Secondly, considering the negative externalities of air pollution and the positive externalities of atmospheric environmental governance, this paper examines the direct impact of FD and GTI on air pollution and its spatial spillover effects from the perspective of intergovernmental competition. Thirdly, focusing on the moderating role of GTI, this paper explores the regulatory effect exerted by GTI on the impact of FD on air pollution and its regional heterogeneity.

**Figure 1:**
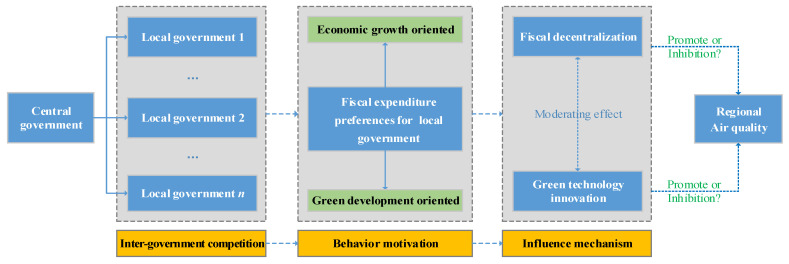
Research framework.

The research framework of this article is shown in Figure 1.

## 2. Mechanism Analysis and Research Hypothesis

The research on environmental issues of fiscal decentralization is called “environmental federalism”. This type of research discusses the generation and governance of environmental issues from the perspective of decentralization [9]. The traditional theory of fiscal decentralization believes that fiscal decentralization, as an institutional arrangement of fiscal division between the central and local governments, helps to increase the enthusiasm of local governments to provide public products according to local conditions. Compared with the central government, local governments can more effectively offer public products based on residents’ preferences and regional needs, thereby helping to improve environmental quality [15]. However, the second-generation fiscal decentralization theory believes that local governments, stimulated by tax revenues, make choices that damage the quality of the regional environment [16]. Many scholars believe FD will aggravate haze pollution by loosening environmental supervision, seizing the market, and other “race to the bottom” behavior [17,18]. Some local governments do not strictly enforce regulations or impose nominal fines on polluters [19], causing pollution to increase. However, some scholars believe that FD is helpful for environmental pollution control [20,21] and has regional heterogeneity. FD may push the two to opposite development strategies for developed and underdeveloped regions. Under the fiscal decentralization system, local governments must develop the economy and improve people’s livelihoods in the meantime. Therefore, it is necessary to coordinate the relationship between economic development and environmental protection [22,23,24], and whether “race to the bottom” or “race to the top” lies in the development orientation of the local government. After the reform of fiscal decentralization, local governments that take economic growth as the development orientation tend to pursue economic interests one-sidedly and ignore environmental protection, engaging in a “race to the bottom”. In contrast, those oriented to green development will promote “competition to the top” through green technology innovation. In addition, the fiscal decentralization of provinces located in different development-oriented regions may have different spatial spillover effects on air pollution [25,26]. Based on the above analysis, this article proposes the following hypotheses:

**Hypothesis** **1a.** *FD has a significant direct impact on air pollution.*

**Hypothesis** **1b.** *The spatial spillover effects of FD on air pollution are significant.*

At present, avoiding a “race to the bottom” through green development is the way to achieve high-quality development. Adhering to the leadership of green development and promoting high-quality economic development with high-level protection of the ecological environment is the key focus of China’s ecological environmental protection policy during the “14th Five-Year Plan” period. Green development is an innovative development, a fundamental change in the development mode, and a breakthrough improvement in development efficiency and quality. It requires the leading role of technology. Therefore, green development is inseparable from green technology innovation [27]. GTI has both “innovation” and “green” characteristics as essential support for realizing green development goals. The current research on GTI focuses on the economic benefits it produces. There are few studies on the environmental benefits of GTI and the specific relationship between it and the environment. However, environmental benefits should be the primary goal of green innovation [28], and most of the current literature does not consider the spatial spillover effects of GTI. Therefore, using the spatial model to further analyze the impact of GTI on environmental pollution based on existing research is necessary. Based on the above analysis, this article proposes the following hypotheses:

**Hypothesis** **2a.** *GTI has a significant direct impact on air pollution.*

**Hypothesis** **2b.** *The spatial spillover effects of GTI on air pollution are significant.*

Previous studies have provided a theoretical framework and empirical tests for analyzing the relationship between FD, GTI, and environmental pollution [15]. Najaf et al. [29] explored the relationship between FD, environmental innovation, and carbon emission reduction. They found short-run causal and unidirectional links running from fiscal decentralization, export diversification, and environment-related technological innovation to carbon emissions. Li et al. [30] believe that environmental decentralization significantly strengthens environmental pollution control due to the improved green innovation ability. However, there are still some shortcomings in the previous literature: First, when analyzing the interaction between fiscal decentralization and innovation behavior, FD is often used as a moderating variable, and it lacks the perspective of GTI as a moderating variable to analyze the impact of its interaction on environmental pollution; second, less consideration is given to regional heterogeneity and spatial spillover effects when studying the regulatory effects; third, is the investigation of innovative behaviors. Most scholars have focused on technological innovations that are conducive to the improvement of the economic efficiency of enterprises. Still, they have insufficient consideration of green technological innovations for pollution reduction. In fact, regional GTI can significantly improve the air environmental quality through optimizing and upgrading the industrial structure and the intensive use of resources [31]. Under the current high-quality regional economic development and green GDP performance evaluation mechanism, regional GTI has a vital demonstration effect in improving air quality and promoting high-quality local economic development. It makes the fiscal expenditures of local governments tend to be green technology research and development, public environmental governance, and other fields. Based on the above consideration, this article believes that GTI can influence the emission reduction effect of the local government’s fiscal decentralized expenditure structure through its demonstration role. Therefore, from the perspective of GTI as a moderating variable, this article proposes the following hypotheses:

**Hypothesis** **3.** *GTI has a significant moderating effect on the relationship between FD and air pollution.*

## 3. Research Design

### 3.1. Spatial Measurement Model Construction

Considering the direct impact of intergovernmental competition on regional air pollution control and its spatial spillover effects, this paper used the SDM model to study the mechanism of FD and GTI on air pollution. This model can examine the spatial relevance of dependent variables and help overcome the spatial influence of random interference items. Therefore, this paper used 30 provinces in China from 2003 to 2018 as a sample to establish the following basic model.

(1) $$PM_{i,t}=\alpha_{0}+\rho \sum_{j=1}^{N}W_{ijt}PM_{i,t}+\alpha_{1}FD_{i,t}+\alpha_{2}GTI_{i,t}+\sum_{k=1}^{4}\beta_{k}X_{i,t}+\theta_{1}\sum_{i\neq j}^{N}W_{ijt}FD_{i,t}+\theta_{2}\sum_{i\neq j}^{N}W_{ijt}GTI_{i,t}+\sum_{k=1}^{4}\lambda_{k}\sum_{i\neq j}^{N}W_{ijt}X_{i,t}+\mu_{i}+\gamma_{t}+\varepsilon_{i,t}$$

In the formula, *i* represents the province; *t* represents the year; *PM* represents the annual average concentration of PM2.5; *FD* represents the fiscal decentralization index; *GTI* represents the green technology innovation; ***X*** represents the control variable that affects the PM2.5 concentration including population density (*POPD*), per capita income level (*PERIN*), urbanization rate (*URBAN*), and rainfall (*RAINF*); $\mu_{i}$ represents individual fixed effects; $\gamma_{t}$ describes time fixed effects; $\varepsilon_{i,t}$ represents random disturbance items.

To test the joint impact of FD and GTI on air pollution, this paper introduces the interaction term between FD and GTI based on model (1), revealing the adjustment of GTI on the reduction effect of FD.

(2) $${\mathrm{PM}}_{i,t}=\alpha_{0}+\rho \sum_{j=1}^{N}W_{ijt}{\mathrm{PM}}_{i,t}+\alpha_{1}FD_{i,t}+\alpha_{2}GTI_{i,t}+\alpha_{3}FD_{i,t}\cdot GTI_{i,t}+\sum_{k=1}^{4}\beta_{k}X_{i,t}+\theta_{1}\sum_{i\neq j}^{N}W_{ijt}FD_{i,t}+\theta_{2}\sum_{i\neq j}^{N}W_{ijt}GTI_{i,t}+\theta_{3}\sum_{i\neq j}^{N}W_{ijt}FD_{i,t}\cdot GTI_{i,t}+\sum_{k=1}^{4}\lambda_{k}\sum_{i\neq j}^{N}W_{ijt}X_{i,t}+\mu_{i}+\gamma_{t}+\varepsilon_{i,t}$$

### 3.2. Spatial Weight Matrix Construction

According to the first law of geography, “Everything is closely connected, but the more adjacent things are connected more closely”, and the spatial weight matrix can describe the degree of association between things. However, considering the spatial characteristics of air pollution, the adjacency weight matrix constructed by Moran [32] cannot truly reflect the economic and social relationships between regions. Therefore, this paper draws on Wang et al. [33] to improve the weight matrix from multiple dimensions and constructs the geographic weight matrix ($W_{ij}^{D}$) and economic weight matrix ($W_{ij}^{E}$) among provinces, respectively. To objectively reflect the comprehensive influence of geographic and economic factors, this paper constructed a comprehensive nested spatial matrix ($W_{ij}$) based on the geographic distance and economic distance matrices. Among them, the matrix $W_{ij}^{D}$ was used for empirical analysis, and the matrix $W_{ij}$ was used for stability testing. Three matrices were constructed as follows:

(3) $$W_{ij}^{D}=1/d_{ij}^{2}$$

(4) $$W_{ij}^{E}=1/\left|Y_{i}-Y_{j}\right|$$

(5) $$W_{ij}=W_{ij}^{D}\times W_{ij}^{E}$$

where $d_{ij}$ represents the distance between two provinces *i* and *j*, and $\left|Y_{i}-Y_{j}\right|$ represents the per capita GDP gap between two provinces. It should be noted that when *i* = *j*, the matrices $W_{ij}^{D}$, $W_{ij}^{E}$, and $W_{ij}$ are equal to 0.

### 3.3. Spatial Autocorrelation Test

To verify whether air pollution has spatial relevance, this paper used the global Moran index to test the degree of spatial relevance. The calculation formula is as follows:

(6) $$I=\frac{\sum_{i=1}^{n}\sum_{j=1}^{n}W_{ij}\left(X_{i}-{\bar{X}}_{i}\right)\left(X_{j}-{\bar{X}}_{j}\right)}{S^{2}\cdot \sum_{i=1}^{n}\sum_{j=1}^{n}W_{ij}}$$

In the formula, $X_{i}$ and $X_{j}$ are the attribute values of area *i* and its surrounding area *j*, respectively; ${\bar{X}}_{i}$ and ${\bar{X}}_{j}$ are the expected average value of $X_{i}$ and $X_{j}$, respectively; $S^{2}=\frac{1}{n}\sum_{i=1}^{n}{\left(X_{i}-{\bar{X}}_{i}\right)}^{2}$ is the sample variance; $W_{ij}$ is the spatial weight matrix; $\sum_{i=1}^{n}\sum_{j=1}^{n}W_{ij}$ is the sum of the spatial weights. After normalizing all elements in the matrix $W_{ij}$, then $\sum_{i=1}^{n}\sum_{j=1}^{n}W_{ij}=n$, and the global Moran index ***I*** can be simplified as:

(7) $$I=\frac{\sum_{i=1}^{n}\sum_{j=1}^{n}W_{ij}\left(X_{i}-{\bar{X}}_{i}\right)\left(X_{j}-{\bar{X}}_{j}\right)}{\sum_{i=1}^{n}{\left(X_{i}-{\bar{X}}_{i}\right)}^{2}}$$

### 3.4. Variable Definition and Data Description

(1) Explained variable. The explained variable in this paper was the degree of air pollution, expressed by the annual average concentration of PM2.5 in each province. The data were sourced from the Atmospheric Composition Analysis Group of Washington University to calculate the global surface PM2.5 concentration.

(2) Core explanatory variables.

(a) Fiscal decentralization (FD). Since the tax system reform, the central government has gradually delegated power to local governments, believing that local governments have a better understanding of the needs and preferences of local residents for public services. It is also the original intention of the fiscal decentralization system. Previous documents have shown that the effectiveness of China’s environmental governance is related to the fiscal decentralization system [34]. The main indicators to measure the degree of FD are the proportion of local revenue and the proportion of expenditure. Based on Chen and Gao [35], this paper selected expenditure indicators and revenue indicators to indicate the degree of fiscal decentralization in which the share of local fiscal revenue was used for empirical testing and the share of local fiscal expenditure was used for stability testing, while the formula for calculating fiscal decentralization was improved by drawing on Gong [36] as follows:

(8) $$FD=\left[\frac{Br_{i}/Pop_{i}}{Br_{i}/Pop_{i}+Br_{c}/Pop_{N}}\right]\times \left[1-\left(\frac{GDP_{i}}{GDP_{N}}\right)\right]$$

Fiscal decentralization is equal to the ratio of local budget revenue per capita to the central and local budget revenue per capita. This indicator measures the disposable budget revenue that local governments have compared with the central government. $Br_{i}$ represents the public budget fiscal revenue of the *i*th province; $Br_{c}$ represents the public budget revenue of the central government; $Pop_{i}$ represents the population size of the *i*th province; $Pop_{N}$ represents the national population size. Equation (6) uses per capita data and filters the impact of the population size and multiplies it by the economic scale reduction factor $\left[1-GDP_{i}/GDP_{N}\right]$ ($GDP_{i}$ is the GDP of the *i*th province, and $GDP_{N}$ is the national GDP) to eliminate the interference of the economic scale on FD.

(b) Green technology innovation (GTI). There have been many studies on the relationship between GTI and environmental pollution. Among them, there are two main methods for measuring GTI: one is to directly measure the intensity of R&D investment, which ignores the “green” feature; the other is to select the green total factor productivity indicator as a proxy variable [12,37], but this weakens the requirement of “technical innovation”. Based on this, this article searched the State Intellectual Property Office according to the “Green List of International Patent Classifications” and obtained provincial-level green patent authorization data to represent green technological innovation.

(3) Control variables. This article also controls several other variables that may affect regional air pollution. Concerning existing studies, this article chose population density (POPD), per capita income (PERIN), urbanization rate (URBAN), and rainfall (RAINF) as control variables. Among them, population density (POPD) was used to reflect the degree of population spatial agglomeration, expressed as the ratio of the population to the area of the administrative area in each province; The per capita income level (PERIN) was used to reflect the level of regional economic development, expressed in terms of the per capita income of the cities and towns in each province; the urbanization rate (URBAN) was used to reflect the process of regional urbanization, expressed in terms of the ratio of the permanent urban population of each province to the total population; rainfall (RAINF) was an essential natural factor in reducing air pollution, and this article expresses the actual rainfall in each province. In addition, this article took the logarithm processing for all variables.

(4) Data source. This paper selected the panel data of 30 provinces in China from 2003 to 2018 as the research object. The source of the rainfall index data was the National Meteorological Science Data Sharing Service Platform, and the remaining index data sources were the “China Statistical Yearbook”, the provincial statistical yearbooks, and the compilation of statistical data for 60 years of New China.

Due to China’s vast territory and uneven spatial development, this study divided the country into eastern, central, and western regions to explore the regional heterogeneity of intergovernmental games. The eastern region includes 11 provinces (cities): Beijing, Tianjin, Hebei, Liaoning, Shanghai, Jiangsu, Zhejiang, Fujian, Shandong, Guangdong, and Hainan; the central region includes 9 provinces: Shanxi, Inner Mongolia, Jilin, Heilongjiang, Anhui, Jiangxi, Henan, Hubei, and Hunan; the western region consists of 10 provinces (cities): Guangxi, Chongqing, Sichuan, Guizhou, Yunnan, Shaanxi, Gansu, Qinghai, Ningxia, and Xinjiang. The descriptive statistics of the related data are shown in Table 1.

## 4. Empirical Test and Result Analysis

### 4.1. Analysis of Spatial Correlation Results

Based on the economic distance matrix, this paper used the global Moran’s I index method to test the spatial correlation. As shown in Table 2, the global Moran’s I index of the average concentration of PM2.5 in various provinces in China from 2003 to 2018 was significantly positive at the level of 1%; that is, there was a significant positive spatial correlation of PM2.5. Most of the Moran’s I index values of FD and GTI indicators were significant. That is, there was also a significant spatial correlation, indicating that the intergovernmental game also needs to consider the interregional interaction between FD and GTI.

**Figure 2:**
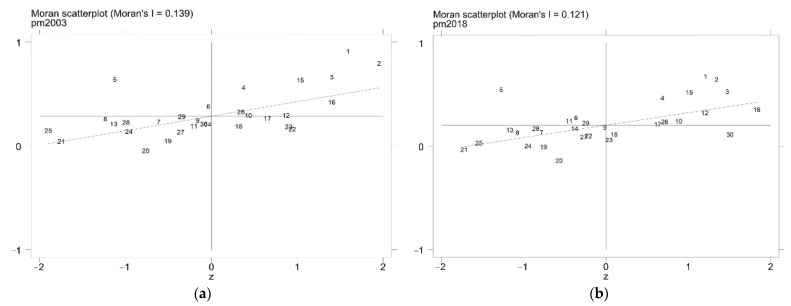
PM2.5 Moran scatter plot. (**a**) 2003. (**b**) 2018.

**Figure 3:**
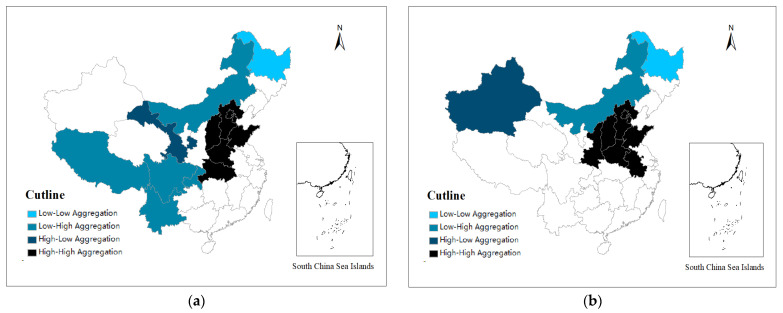
PM2.5 LISA cluster map. (**a**) 2003. (**b**) 2008.

**Figure 4:**
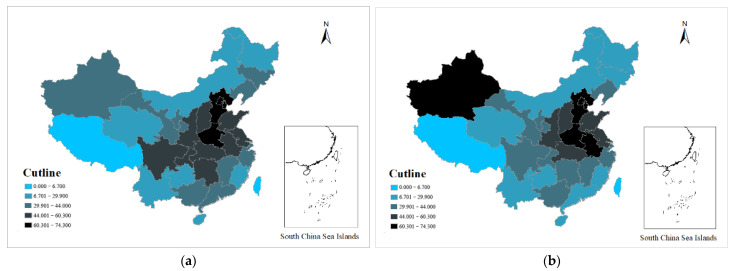
PM2.5 distribution map. (**a**) 2003. (**b**) 2018.

To further study the local spatial correlation of the explained variable PM2.5, this paper drew a Moran scatter plot and a LISA plot to analyze the clustering characteristics. It can be seen from Figure 2 that PM2.5 also had a significant positive spatial correlation (Table A1 in Appendix A shows the province corresponding to each number). Figure 3 also show that PM2.5 had a significant high–high aggregation phenomenon. It shows that areas with severe air pollution had higher levels of air pollution in their neighboring areas, and the high–high concentration areas were mostly the Beijing–Tianjin–Hebei region. It can also be seen from the distribution maps of PM2.5 in Figure 4 that although the range of severe air pollution was shrinking, the Beijing–Tianjin–Hebei region and the Fenwei Plain were still heavily polluted.

### 4.2. Optimal Model Selection and Testing

Anselin [38] pointed out the existence of a spatial correlation between the economic behavior of a region and that of other regions and constructed the spatial error model (SEM), spatial lag model (SAR), and spatial Durbin model (SDM) based on the idea of spatial economic units. These models examine the spatial dependence and spillover characteristics of the independent variable spatial lag term, the dependent variable spatial lag term, and the spatial dependence on geography when the independent and dependent variables’ spatial lag terms co-exist, respectively. Therefore, to determine the optimal form of the regression model, this paper performed LM tests on the model according to the method proposed by Anselin.

The test results in Table 3 show that the four nationwide model indicators significantly rejected the null hypothesis. In terms of the eastern, central, and western regions, except for the robust LM (Lag) statistic, the rest of the statistics significantly reject the original hypothesis. It shows that there were spatial correlation terms and spatial lag terms in the set models, and the spatial Durbin model (SDM) can more comprehensively integrate the spatial correlation term and the spatial lag term. Therefore, the spatial Durbin model (SDM) was initially chosen in this paper. To further determine whether the SDM model would degenerate into a spatial lag model (SAR) and a spatial error model (SEM), this paper continued the LR and Wald tests, and the results are shown in Table 4. The LR and Wald test results were both significant at the 1% level; thus, the SDM model was finally selected in this article.

Finally, the Hausman test was needed to determine whether it was a fixed-effects model or a random-effects model. In Table 5, the Hausman test results show that the national and regional results significantly reject the null hypothesis; therefore, the fixed-effects model should be selected. After determining the fixed-effects model, we need to use a mixed significance test to determine further which fixed-effects model to use. The joint significance test results in Table 6 show significant individual and temporal differences in the variables. Thus, considering the effects of intra-temporal factors and individual regional differences, the model was finally set as an individual-time dual-fixed SDM in this paper.

### 4.3. Result Analysis and Discussion

According to the basic model (1), this paper examined the direct impact and spatial spillover effects of FD and GTI on regional air pollution. The results are shown in the first and third columns of Table 7. From the test results in the first column of Table 7, it can be seen that the local fiscal decentralization significantly suppressed the level of local air pollution at a confidence level of 1%, with a coefficient of −0.214. The root causes were two-fold: on the one hand, the increase in FD allows local governments to have greater financial autonomy, and with the society’s call for environmental governance and the implementation of the concept of green GDP performance, local governments have become increasingly inclined to increase financial investment in public environmental products; on the other hand, according to the “voting with feet” mechanism of Tiebout [39], the FD system can incentivize local governments to provide better environmental quality to obtain more citizen votes [40]. In addition, local GTI significantly curbed local air pollution levels up to a 1% confidence level with a coefficient of −0.057. Green technological innovation promotes the optimization and upgrading of the industrial structure and energy structure at a macrolevel, thereby reducing the emission of air pollutants [41]. At the microlevel, GTI involves technological innovation of production and governance [27,42]. The former mainly achieved the intensive use of resources and the total emission reduction of pollutants through the green upgrading of production processes, while the latter achieved the improvement of regional air quality through the upgrading of pollution control technologies and pollution treatment facilities in production. In summary, Hypothesis 1a and Hypothesis 2a are verified.

The third column in Table 7 are the test results of the spatial spillover effect of FD and GTI on air pollution. The results show that the spatial spillover effect of FD on air pollution was negative but not significant; therefore, Hypothesis 1b was not verified. The root cause was that due to the regional differences in resource endowments, industrial structure, and development demands, under the current administrative barriers and institutional constraints, the spatial spillover effect of FD was not significant. In contrast, the spatial spillover effect of GTI on air pollution was significantly negative at the 1% confidence level. That is, an increase in the level of GTI in neighboring areas will significantly reduce the concentration of local air pollution, and this shows that Hypothesis 2b was verified. It may be due to the positive externality of air pollution control. After the neighboring area has improved the local air quality through GTI, the area has shared the results of neighboring air pollution control due to the fact of air spills and has taken a ride on the neighboring air pollution control [43,44]. That is, the neighboring provinces of high-GTI provinces rarely choose to improve air pollution through GTI and mostly want to share the green patents and environmental management achievements of high-GTI areas [45].

With regard to the correlation between FD and GTI behavior, this paper introduces the interaction terms between FD and GTI based on the benchmark model (1). The relevant estimation results are shown in the second and fourth columns of Table 7. Overall, the direction of the impact of FD and GTI on regional air pollution has not changed. Among them, green innovation significantly suppressed the degree of air pollution at the 1% confidence level with a coefficient of −0.107, and its spatial spillover effect was −0.647, which is significantly larger than the local direct effect. In contrast, the local direct and spatial spillover effects of fiscal decentralization on air pollution were both negative but not significant. The test results of the interaction terms between the two (the second column of Table 7) show that the estimated coefficient was negative and significant at the 1% confidence level. This indicates that the local fiscal decentralization under the guidance of green innovation behavior had positively promoted local air quality; therefore, Hypothesis 3 was verified. The root causes lie in the context of the green GDP performance evaluation system and the competition and incentives among local governments; the demonstration and leading role of green technological innovation will cause the provincial government’s fiscal decentralization expenditure structure to be more inclined toward green-biased technological innovation activities, which will be more helpful for improving the quality of the regional air environment. At the same time, due to the positive externalities of GTI and air governance, the higher the regional GTI, the demonstration effect will stimulate other regions’ financial investment in green technology research and development and ecological environment governance. This results in the innovation compensation effect offsets the compliance cost caused by environmental protection, thereby affecting the environmental governance performance of surrounding areas [46].

### 4.4. Regional Heterogeneity Analysis

According to the analysis mentioned above, there is a strong correlation between FD, GTI, and environmental pollution in the global space. However, due to the unbalanced status of regional development, the local space may show an atypical situation that is different or utterly contrary to the global space. Therefore, this article discusses the regional heterogeneity between FD, GTI, and air pollution. The corresponding test results are shown in Table 8.

(1) the local direct effects of FD and GTI on air pollution

According to Table 8 (columns 2, 4, and 6), FD significantly suppressed air pollution in the eastern region at a 1% confidence level with a factor of −0.990, exacerbated air pollution in the western region, and had no significant impact on the PM2.5 in the central region. This means that there were significant regional differences in the effects of FD on air pollution. As the most economically developed region in China, increased FD in the eastern region was conducive to achieving air pollution control by local governments. Still, the central and western regions were just the opposite. The reason for such differences may be that the eastern region was the most developed economically, where environmental pollution is a prominent issue, and local governments are compelled to address the issue of carbon emissions [27]. Compared to the eastern region, economic development in the central and western regions is limited, and environmental pollution is considerably less compared to their eastern counterpart as shown in Figure 3. Meanwhile, awareness of environmental governance and the level of economic development is higher in the eastern region than in the central and western regions, making FD provide greater financial freedom to the eastern provinces to improve the environmental quality effectively [26].

As presented in Table 8 (columns 2, 4, and 6), GTI significantly exacerbated the air pollution in the eastern region and suppressed the air pollution in the central and western regions, which also means that there were significant regional differences. Although GTI aggravated air pollution in the eastern region to a lesser extent, it was also worthy of attention. We believe this may be due to the environmental rebound effect in the east. The environmental rebound effect refers to the fact that the GTI behavior reduced the unit production cost of the product and the resource utilization efficiency, which will make people expect the price of resources to fall, thus increasing the demand for resources [47]. Moreover, the improvement in resource utilization efficiency will increase economic output, stimulate investment, further increase the demand for resources, and ultimately increase environmental pollution [48]. After green technology innovations in the eastern region, they tasted the “sweetness” of GTI. To maximize the benefits, increasing investment in GTI resulted in a waste of costs and resources and ultimately increased pollution. In the western region, GTI is in its infancy. The initial results of GTI achieved a certain level of emission reduction, and as a result, air pollution was suppressed.

(2) the spatial spillover effects of FD and GTI on air pollution

From the results of Model 1 in Table 8 (columns 2, 4, and 6), it can be concluded that: (a) In the central and western regions, the spatial spillover effect of FD on air pollution was significant at the 1% confidence level, and when the degree of FD in neighboring provinces increased by one unit, the local air quality deteriorated by 1.496 and 0.471, respectively. This may be due to the only-economic-growth development orientation and the “bottom competition” among provinces in the central and western regions [49,50]. That is to say, the improvement in FD gives local governments a more relaxed institutional environment, which makes them neglect environmental pollution control to stimulate regional economic development. On the contrary, the spatial spillover effect of FD on air pollution was not significant in the eastern region. (b) The spatial spillover effects of GTI in the central and western regions on air pollution were both negatively significant at the 1% confidence level, while in the eastern region, it had no significant impact. This shows that when the GTI in the adjacent areas of the central and western regions increases, the level of air pollution in the region will decrease. This may be because the public attributes of environmental products determine their significant externalities, which gives local governments in the central and western regions “free-riding” motivation in environmental governance [51]. In contrast, in the economically developed and more polluted eastern region, the spatial spillover effect of GTI on air pollution was not apparent due to the central government’s pressure on local environmental supervision and its own need for green development.

(3) the moderating effect of GTI on the relationship between FD and air pollution

From the results of Model 2 in Table 8 (columns 2, 4, and 6), we can conclude: (a) GTI in the west played a significant negative moderating effect on the direct effect of FD on air pollution at a 1% confidence level with a coefficient of −0.119. This means that an increase in local GTI raised the impact of local FD on improving air quality. (b) The GTI in the eastern region played a significant positive role in the direct effect of FD on air pollution with a coefficient of 0.107, which means that an increase in local GTI will weaken the environmental effect of local FD at the 5% confidence level. In terms of different regions, the effect of GTI on the relationship between FD and air pollution through demonstration effects in the eastern region was not satisfactory, which may be due to the impact of the environmental rebound effect mentioned above. Under the leading role of GTI, the eastern region’s fiscal revenues continue to tilt towards green technological innovation. Blind and excessive investment has resulted in increased costs and wasted resources, which eventually backfired and increased air pollution [52]. In contrast, the western region was “competing to the top” under the leading role of GTI. The higher the level of GTI in a province, the demonstration effect will stimulate other provinces to invest in green technology research and development and ecological environment governance. In addition, the need to develop infrastructure and the relatively small degree of FD in the western inland regions limit its fiscal freedom to maintain an appropriate and reasonable level of investment in GTI. As a result, the air quality in the west region will eventually improve.

### 4.5. Model Robustness Test

To strengthen the credibility of the empirical results, the economic geography matrix was replaced by a geographic distance matrix for robustness testing. The empirical results of the model are shown in Table 9. At the national level, local FD and GTI still significantly curbed air pollution, indicating that the FD and GTI had a positive effect on improving local air quality; FD and GTI in neighboring areas also had a restraining effect on local air pollution. The local fiscal decentralization decreased the local air pollution with the moderating effect of GTI at the 1% confidence level. From a regional perspective, the impact of the core explanatory variables on the explained variables was basically the same as the previous article. Overall, the experimental results of this article are robust.

## 5. Conclusions and Policy Implications

### 5.1. Main Conclusions

Based on the panel data of 30 provinces in China from 2003 to 2018, this paper used the SDM model to examine the direct effect of FD and GTI on regional air pollution and its spatial spillover effect. We also focused on GTI behavior and analyzed its moderating effect on the emission reduction effect of FD and its regional heterogeneity. The main research conclusions are as follows.

Regional air pollution had a significant spatial positive correlation, and Beijing–Tianjin–Hebei and Fenwei Plain showed high agglomeration characteristics. Across the country, the increase in FD and GTI in various provinces had a significant inhibitory effect on local air pollution, and GTI had a significant spatial spillover effect on air pollution reduction. That is, GTI in neighboring areas will significantly inhibit local air pollution. The demonstrative effect of GTI encourages the “race to the top” between governments in the FD expenditure structure. The demonstrative effect induces local government FD to be more inclined to green-biased technological innovation activities, which is more helpful in improving the quality of the regional air environment. From a regional perspective, there were significant regional differences in the impact of FD and GTI on air pollution. Due to the low level of economic development in the western region, its economic development fiscal expenditures squeezed out environmental governance fiscal expenditures, resulting in FD promoting local air pollution with noticeable positive spillover effects. However, under the leading role of GTI, the western region will compete on top of each other. Finally, the appropriate fiscal tilt for GTI improved air quality. The eastern regions had a better economic foundation, and FD has given local governments greater enthusiasm and autonomy in pollution control, thereby helping to improve regional air quality. In contrast, GTI had a significant impact on air pollution. Compared with the west, GTI in the eastern region had a more significant impact on air pollution, but the demonstration effect of GTI in the eastern region did not allow FD to play a positive role in reducing emissions. The reason was that the eastern region had a relatively high level of GTI and low pollution reduction potential, resulting in high marginal GTI costs and marginal emission reduction costs. In turn, it transforms its fiscal expenditure structure into an economically efficient industry, eventually leading to an increase in pollution emissions and an environmental rebound effect.

### 5.2. Policy Implications

According to the above conclusions, this paper proposes the following policy recommendations.

It is necessary to make full use of the institutional advantages of FD, optimize the expenditure structure of FD, and improve regional air quality in accordance with local conditions. For the eastern region, where FD is conducive to improving air quality, local governments should give full play to the information advantages in providing public goods, optimizing the expenditure structure and intensity of FD, and balancing economic development and air environmental protection. For the less economically developed central and western regions, the central government should strengthen the regulation of the local environment along with the fiscal decentralization system, provide appropriate intervention and assistance, and increase the motivation of local governments in environmental governance.

The government should pay attention to the demonstration role of GTI and try to avoid environmental rebound effects. Properly carrying out GTI is conducive to the improvement of environmental quality, and its demonstration effect can drive other regions to increase financial investment in green technology research and development, thereby promoting regional environmental improvement. However, as the saying goes, too much water drowned the miller. If it is too dependent on green technological innovation and blindly invests in production, it will eventually cause an environmental rebound effect, harming the environment. Therefore, along with improving the level of green technology innovation, we should also improve the access conditions for new enterprises, regularly eliminate backward production capacity, strengthen the supervision of pollution emissions, and promote green development in a scientific and orderly manner. To this end, the government should strengthen the construction of the green development standard system, continuously improve the long-term mechanism conducive to the development of green technology as well as the pollution supervision mechanism and create a suitable environment for technological innovation.

Local governments should gradually build a green performance evaluation mechanism to break down the administrative barriers to cooperative governance of air pollution and bring into play the spatial spillover effects of green technological innovation, so as to achieve high-quality regional economic development and continuous improvement of environmental quality. Especially for highly polluted regions, such as the Beijing–Tianjin–Hebei region and the Fenwei Plain, it is imperative to establish a green GDP-oriented performance evaluation mechanism [24], gradually incorporate the supply of public products such as environmental quality and environmental governance investment into the performance target evaluation system and strengthen the environmental governance accountability mechanism, such as out-of-service audits. Secondly, the government should encourage enterprises to carry out independent innovation of dedicated emission reduction technologies in the form of R&D subsidies and encourage the application of general-purpose green technologies among governments through financial subsidies or tax incentives, so as to give full play the spatial spillover effect of green technologies. Furthermore, it is necessary to break down the administrative barriers to “territorial control” of air pollution to avoid the phenomenon of a “race to the bottom” competition among governments and gradually establish a joint prevention mechanism and coordinated policy for air pollution control and adopt market-based measures, such as emission rights trading and pollution tax reform, to resolve the negative externalities of air pollution. For example, the Beijing–Tianjin–Hebei region and the Fenwei Plain are both highly polluted areas. Therefore, they should enhance regional emergency linkages in heavily polluted weather, exchange pollution management experience, and jointly conduct green innovation R&D to crack outstanding environmental problems.

## Figures and Tables

**Table 1:** Descriptive statistics of the variables.

**Nationwide**						**West Region**					
**Variable**	**Mean**	**SD**	**Minimum**	**Maximum**	**N**	**Variable**	**Mean**	**SD**	**Minimum**	**Maximum**	**N**
ln(PM_it_)	3.79	0.40	2.73	4.51	480	ln(PM_it_)	3.73	0.34	2.75	4.33	160
ln(FD_it_)	−0.81	0.27	−1.38	−0.20	480	ln(FD_it_)	−0.95	0.20	−1.38	−0.56	160
ln(GTI_it_)	4.93	1.78	0.00	8.83	480	ln(GTI_it_)	4.05	1.70	0.00	7.30	160
ln(POPD_it_)	11.05	1.23	4.90	13.78	480	ln(POPD_it_)	10.63	1.01	7.50	12.68	160
ln(PERIN_it_)	10.69	1.53	6.22	14.39	480	ln(PERIN_it_)	11.46	1.31	9.28	14.39	160
ln(URBAN_it_)	2.73	0.06	2.50	2.87	480	ln(URBAN_it_)	2.66	0.16	2.21	2.82	160
ln(RAINF_it_)	0.60	0.54	−0.43	1.92	480	ln(RAINF_it_)	0.46	0.49	−0.43	1.25	160
**Central Region**						**East Region**					
**Variable**	**Mean**	**SD**	**Minimum**	**Maximum**	**N**	**Variable**	**Mean**	**SD**	**Minimum**	**Maximum**	**N**
ln(PM_it_)	3.80	0.41	2.73	4.51	144	ln(PM_it_)	3.84	0.43	2.75	4.51	176
ln(FD_it_)	−0.94	0.20	−1.34	−0.51	144	ln(FD_it_)	−0.59	0.24	−1.10	−0.20	176
ln(GTI_it_)	4.78	1.37	1.10	7.37	144	ln(GTI_it_)	5.84	1.71	0.69	8.83	176
ln(POPD_it_)	11.23	1.04	8.16	13.47	144	ln(POPD_it_)	11.30	1.46	4.90	13.78	176
ln(PERIN_it_)	10.30	1.60	6.22	13.14	144	ln(PERIN_it_)	12.08	0.95	9.48	14.39	176
ln(URBAN_it_)	2.74	0.05	2.62	2.83	144	ln(URBAN_it_)	2.75	0.06	2.54	2.87	176
ln(RAINF_it_)	0.49	0.50	−0.40	1.34	144	ln(RAINF_it_)	0.81	0.54	−0.32	1.92	176

**Table 2:** Moran’s I index of core variables.

Year	PM		FD		GTI	
	*I*	*p*-Value *	*I*	*p*-Value *	*I*	*p*-Value *
2003	0.139 ***	0.000	0.019 *	0.066	−0.053	0.306
2004	0.074 ***	0.004	0.019 *	0.066	−0.032	0.475
2005	0.091 ***	0.001	0.016 *	0.076	0.031 **	0.036
2006	0.108 ***	0.000	0.034 **	0.029	−0.003	0.192
2007	0.093 ***	0.001	0.033 **	0.032	0.056 ***	0.008
2008	0.094 ***	0.001	0.028 **	0.045	0.006	0.142
2009	0.114 ***	0.000	0.036 **	0.032	0.114 ***	0.000
2010	0.078 ***	0.003	0.033 **	0.041	0.062 ***	0.006
2011	0.100 ***	0.000	0.024 *	0.068	0.015 *	0.097
2012	0.080 ***	0.002	0.028 *	0.057	−0.012	0.272
2013	0.149 ***	0.000	0.042 **	0.026	−0.052	0.316
2014	0.159 ***	0.000	0.036 **	0.036	−0.058	0.269
2015	0.173 ***	0.000	0.023 *	0.072	−0.087 *	0.081
2016	0.163 ***	0.000	0.030 *	0.049	−0.096 *	0.053
2017	0.138 ***	0.000	−0.008	0.244	−0.086 *	0.084
2018	0.121 ***	0.000	−0.029	0.445	−0.093 *	0.059

*** *p* < 0.01, ** *p* < 0.05, and * *p* < 0.10.

**Table 3:** LM test results.

Area	Nationwide	East	Central	West
LM test (Error)	829.963 ***	93.101 ***	83.259 ***	50.013 ***
Robust LM (Error)	281.414 ***	84.360 ***	77.013 ***	45.801 ***
LM test (Lag)	553.216 ***	8.806 ***	6.746 ***	5.677 **
Robust LM (Lag)	4.667 **	0.065	0.499	1.464

*** *p* < 0.01, ** *p* < 0.05.

**Table 4:** LR test and Wald test results.

Area	Nationwide	East	Central	West
LR test (SDM and SAR)	54.77 ***	32.27 ***	43.82 ***	43.30 ***
LR test (SDM and SEM)	67.75 ***	32.42 ***	52.68 ***	51.54 ***
Wald test	56.06 ***	34.01 ***	48.32 ***	41.19 ***
	70.05 ***	31.42 ***	49.25 ***	30.25 ***

*** *p* < 0.01.

**Table 5:** Hausman test results.

Area	Nationwide	East	Central	West
Hausman test	34.72 ***	90.56 ***	108.67 ***	18.05 *

Z-statistics are in parentheses. *** *p* < 0.01, * *p* < 0.10.

**Table 6:** Joint significance test results.

Area	Nationwide	East	Central	West
LR test (both and individual)	55.78 ***	38.80 ***	53.97 ***	36.86 ***
LR test (both and time)	975.23 ***	236.08 ***	52.50 ***	205.88 ***

*** *p* < 0.01.

**Table 7:** Spatial measurement results based on economic geographic matrix.

Variable	Local Direct Effect		Spatial Spillover Effect	
	(1)	(2)	(1)	(2)
ln(FD)	−0.214 ***	−0.012	−1.451	−0.931
	(−2.43)	(−0.13)	(−1.16)	(−0.81)
ln(GTI)	−0.057 ***	−0.107 ***	−0.678 **	−0.647 **
	(−3.55)	(−5.19)	(−2.53)	(−2.14)
ln(FD) × ln(GTI)		−0.071 ***		−0.328
		(−4.19)		(−1.26)
ln(POPD)	0.074 ***	0.065 ***	0.628 **	0.433 *
	(4.79)	(4.26)	(2.43)	(1.88)
ln(PERIN)	0.407 **	0.361 **	4.142 *	3.634
	(2.52)	(2.32)	(1.66)	(1.43)
ln(URBAN)	0.044	−0.072	0.180	−1.577
	(0.51)	(−0.89)	(0.12)	(−1.17)
ln(RAINF)	−0.035	−0.022	−1.420 **	−1.252 **
	(−0.79)	(−0.47)	(−2.32)	(−2.15)

Z-statistics are in parentheses. *** *p* < 0.01, ** *p* < 0.05, * *p* < 0.10.

**Table 8:** Results of regional heterogeneity estimation.

**Variable**	**Local Direct Effect**					
	**West**		**Central**		**East**	
	**(1)**	**(2)**	**(1)**	**(2)**	**(1)**	**(2)**
ln(FD)	0.034	0.490 ***	−0.147 ***	0.300	−0.654 ***	−0.990 ***
	(0.21)	(2.61)	(−1.07)	(0.91)	(−4.02)	(−3.42)
ln(GTI)	−0.080 ***	−0.170 ***	−0.148 ***	−0.261 ***	−0.002	0.089 **
	(−3.50)	(−5.01)	(−3.80)	(−2.88)	(−0.07)	(2.31)
ln(FD) × ln(GTI)		−0.119 ***		−0.115		0.107 **
		(−3.69)		(−1.54)		(2.14)
ln(POPD)	0.028 *	0.037 **	0.096 ***	0.141 ***	0.102 **	0.069 *
	(1.67)	(2.25)	(2.86)	(3.68)	(2.21)	(1.75)
ln(PERIN)	0.306	0.422 *	−0.290	−0.166	0.511 **	0.619 ***
	(1.31)	(1.92)	(−0.67)	(−0.42)	(2.08)	(4.57)
ln(URBAN)	−0.223 **	−0.152	0.207	0.489 *	0.603 **	0.792 **
	(−2.10)	(−1.54)	(0.76)	(1.80)	(2.19)	(2.50)
ln(RAINF)	−0.006	0.017	−0.124	−0.038	−0.064	−0.067
	(−0.06)	(0.17)	(−1.56)	(−0.44)	(−1.18)	(−1.21)
**Variable**	**Spatial Spillover Effect**					
	**West**		**Central**		**East**	
	**(1)**	**(2)**	**(1)**	**(2)**	**(1)**	**(2)**
ln(FD)	1.457 ***	3.430 ***	0.815	4.787 ***	−1.067	−3.772
	(4.15)	(5.15)	(1.43)	(3.20)	(−0.82)	(−1.34)
ln(GTI)	−0.129	−0.472 ***	−0.605 ***	−1.496 ***	−0.479 **	0.293
	(−1.51)	(−3.41)	(−3.40)	(−3.96)	(−2.37)	(0.92)
ln(FD) × ln(GTI)		−0.508 ***		−0.850 ***		0.767 *
		(−2.97)		(−2.88)		(1.68)
ln(POPD)	0.152 ***	0.120 ***	0.179	0.354 ***	0.341 **	0.294
	(2.60)	(2.68)	(1.42)	(2.64)	(1.79)	(1.48)
ln(PERIN)	0.01	−0.628 *	−0.087	0.202	−1.567	−1.247 **
	(0.03)	(−1.82)	(−0.05)	(0.14)	(−0.98)	(−2.51)
ln(URBAN)	−0.705	−0.428	−1.637	−1.357	2.244	3.715
	(−1.46)	(−1.09)	(−1.43)	(−1.25)	(1.12)	(1.49)
ln(RAINF)	−0.556	−0.145	−1.014 ***	−0.717 **	−1.014 ***	−0.758 **
	(−1.60)	(−0.48)	(−2.82)	(−2.11)	(−2.96)	(−2.54)

Z-statistics are in parentheses. *** *p* < 0.01, ** *p* < 0.05, * *p* < 0.10.

**Table 9:** Results of stability test.

**Variables**	**Local Direct Effect**							
	**Nationwide**		**West**		**Central**		**East**	
	**(1)**	**(2)**	**(1)**	**(2)**	**(1)**	**(2)**	**(1)**	**(2)**
ln(FD)	−0.216 **	−0.038	0.565 **	1.036 ***	−0.310 **	−0.182	−0.586 ***	−0.750 ***
	(−2.49)	(−0.41)	(1.77)	(2.74)	(−2.42)	(−0.58)	(−4.49)	(−4.90)
ln(GTI)	−0.057 ***	−0.103 ***	−0.056 *	−0.184 ***	−0.098 ***	−0.131	−0.024	0.047 *
	(−3.56)	(−5.15)	(−1.86)	(−2.87)	(−2.96)	(−1.56)	(−0.95)	(1.71)
ln(FD) × ln(GTI)		−0.067 ***		−0.108 *		−0.036		0.060 **
		(−3.99)		(−1.95)		(−0.52)		(2.15)
ln(POPD)	0.079 ***	0.068 ***	0.048 *	0.089 ***	0.076 **	0.100 ***	0.085 **	0.063
	(4.98)	(4.29)	(1.76)	(3.28)	(2.39)	(2.76)	(2.00)	(1.49)
ln(PERIN)	0.434 ***	0.412 **	1.446 ***	1.688 ***	−0.264	−0.213	0.546 ***	0.443 **
	(2.57)	(2.52)	(3.34)	(4.39)	(−0.68)	(−0.59)	(2.67)	(2.09)
ln(URBAN)	0.029	−0.076	1.017 ***	1.063 ***	0.257	0.479 *	0.423 **	0.676 ***
	(0.33)	(−0.92)	(7.32)	(8.73)	(0.97)	(1.75)	(2.21)	(3.16)
ln(RAINF)	−0.035	−0.022	−0.025	0.027	−0.065	−0.014	−0.029	−0.040
	(−0.78)	(−0.47)	(−0.33)	(0.36)	(−0.86)	(−0.18)	(−0.59)	(−0.76)
**Variable**	**Spatial Spillover Effect**							
	**Nationwide**		**West**		**Central**		**East**	
	**(1)**	**(2)**	**(1)**	**(2)**	**(1)**	**(2)**	**(1)**	**(2)**
ln(FD)	−1.287	−0.852	3.891 ***	5.674 ***	0.418	1.930 ***	−0.161	−0.380
	(−1.03)	(−0.71)	(3.52)	(4.98)	(1.61)	(2.83)	(−0.32)	(−0.82)
ln(GTI)	−0.660 **	−0.586 *	0.032	−0.382 **	−0.238 ***	−0.595 ***	−0.172 **	−0.105
	(−2.44)	(−1.82)	(0.41)	(−2.51)	(−2.85)	(−3.33)	(−2.24)	(0.99)
ln(FD) × ln(GTI)		−0.287		−0.428 ***		−0.329 **		0.155
		(−1.08)		(−3.21)		(−2.25)		(1.35)
ln(POPD)	0.745 **	0.502 *	−0.279 ***	−0.145 **	0.079	0.135 *	0.171	0.113
	(2.41)	(1.79)	(−3.58)	(2.13)	(1.2)	(1.90)	(1.62)	(1.11)
ln(PERIN)	5.204 *	5.156 *	1.145	0.726	−0.208	−0.397	−0.615	−1.090
	(1.77)	(1.69)	(0.70)	(0.50)	(−0.23)	(−0.46)	(−0.97)	(−1.45)
ln(URBAN)	−0.280	−1.684	−0.357	−0.098	−0.831	−0.664	0.532	1.159
	(−0.18)	(−1.22)	(−0.73)	(−0.25)	(−1.46)	(−1.11)	(0.62)	(1.42)
ln(RAINF)	−1.606 **	−1.534 **	−0.478 *	−0.308	−0.479 ***	−0.379 **	−0.342 **	−0.359 **
	(−2.23)	(−2.21)	(−1.89)	(−1.33)	(−2.87)	(−2.17)	(−2.96)	(−2.39)

Z-statistics are in parentheses. *** *p* < 0.01, ** *p* < 0.05, * *p* < 0.10.

## Data Availability

Data are available from the authors upon reasonable request as the data need further use.

## Appendix A

**Table A1:** Provinces corresponding to the numbers in the Moran scatter chart.

No.	Province/City	No.	Province/City	No.	Province/City
1	Beijing	11	Zhejiang	21	Hainan
2	Tianjing	12	Anhui	22	Chongqing
3	Heibei	13	Fujian	23	Sichuan
4	Shanxi	14	Jiangxi	24	Guizhou
5	Nei Menggu	15	Shandong	25	Yunnan
6	Liaoning	16	Henan	26	Shanxi
7	Jilin	17	Hubei	27	Gansu
8	Hei Longjiang	18	Hunan	28	Qinghai
9	Shanghai	19	Guangdong	29	Ningxia
10	Jiangsu	20	Guangxi	30	Xinjiang
